# Dynamic weighted ensemble model for predictive optimization in green sand casting: Advancing industry 4.0 manufacturing

**DOI:** 10.1016/j.mex.2025.103393

**Published:** 2025-05-25

**Authors:** Rajesh V․ Rajkolhe, Dr. Sanjay S․ Bhagwat, Dr. Priyanka V․ Deshmukh

**Affiliations:** a Babasaheb Naik College of Engineering, Pusad, Maharashtra, India; b Department of Mechanical Engineering, Babasaheb Naik College of Engineering, Pusad, Maharashtra, India; c Department of Artificial Intelligence and Machine Learning, Symbiosis Institute of Technology, Pune Campus, Symbiosis International (Deemed University), Pune, Maharashtra, India

**Keywords:** Green sand casting, Dynamic weights ensemble, Industry 4.0, Machine learning, Predictive modeling, Dynamic weighted ensemble model

## Abstract

This research presents an enhanced predictive model for green sand casting, designed to tackle the nonlinear complexities arising from interdependent process parameters. Casting defects substantially affect product quality and rejection rates, making accurate prediction vital. To overcome the limitations of individual machine learning models and static ensemble strategies, a novel Dynamic Weighted Ensemble (DWE) model is introduced. The model dynamically allocates weights to top-performing algorithms based on their 10-fold cross-validated RMSE, ensuring robust and adaptive prediction performance.

Five models—Linear Regression, Ridge Regression, Decision Tree, Random Forest, and Gradient Boosting—were evaluated over ten folds. Based on their average RMSE values, the top three models (Gradient Boosting: 8.25, Ridge Regression: 8.30, Linear Regression: 8.31) were selected. The DWE model, applied on five-fold unseen test data using dynamically computed weights, achieved an average RMSE of 8.07. This reflects a 2.1 % improvement in RMSE and a 2.3 % increase in prediction accuracy over the best individual model. The gains were statistically significant (*p* < 0.05) based on paired *t*-test analysis, confirming that DWE offers superior prediction consistency.

The proposed DWE model supports real-time optimization in green sand casting, helping reduce defects and improve quality outcomes. It aligns with Industry 4.0 objectives by promoting automated, data-driven decision-making and smart manufacturing practices.

• Proposed a novel Dynamic Weighted Ensemble (DWE) model for improved defect prediction in green sand casting.
• Achieved a 2.1 % RMSE reduction and 2.3 % accuracy gain over the best individual model with statistical significance (*p* < 0.05).
• Supports Industry 4.0 by enabling real-time, data-driven decision-making in smart manufacturing.

## Graphical abstract

**Figure:**
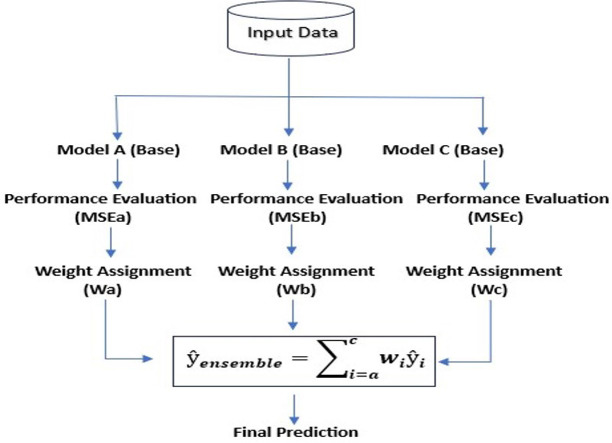

Specifications table

**Table:** 

Subject area:	Computer Science
More specific subject area:	Ensemble Learning
Name of your method:	Dynamic weighted ensemble model
Name and reference of original method:	None
Resource availability:	https://GitHub.com/rvrajkolhe/Casting_Data

## Background

Casting is widely used in manufacturing industries especially in automobiles, aircrafts and heavy machines industries. It is estimated and aptly noted that “we are never more than 3 m away from a metal casting,” a declaration made by the American Foundry Society [1]. Out of many casting techniques, green sand casting has been very appreciated mostly and outstanding in terms of costs. However, casting inherently includes certain variabilities and flaws in the castings still remain difficult to eliminate even when the process parameters of the casting are maintained at optimal levels dominant in the industry [2]. These defects are characterized by high rejection rates hence incurring high material and financial losses. This requires a tool to predict acceptance rates as a function of the process parameters and there is currently none [3].

Such challenges can however be managed by Artificial Intelligence (AI), more specifically by the approach of machine learning. Machine learning still allows systems to influence their decisions and function based on data without the need for programmers to code it in [4]. Nevertheless, these models require high quality, structured, and large data to perform [5]. The datasets employed in this research are structured and include process parameters as the input variables and the defect rates as output variables. Indeed, due to some of the mentioned limitations in data availability, techniques for synthetically generating data are used to extend the dataset by generating statistically similar data for effective training and testing of the models [6,7].

Supervised machine learning algorithms including Linear Regression, Decision Tree Regression, Random Forest Regression, Ridge regression and Gradient Boosting are used in this study to analyse casting acceptance rates. The rising models are appraised based on regression measures such as Mean Squared Error (MSE), Root Mean Squared Error (RMSE), as well as R² score [[8], [9], [10]]. Although single models share distinct potential, classical ensemble methods with fixed assigning, for example, 50 %, 30 %, 20 % of the space to models a, b, and c respectively, do not take advantage of the model strengths in an optimal and adaptive manner. To overcome this limitation, a dynamic weighted ensemble method is put forward. In this approach, for the top 3 best individual models, their weights are calculated weight based on the testing results thereby make the ensemble learn from the best aspects of each model [[11], [12], [13]].

The produced outcomes confirm that the dynamic weighted ensemble model is superior to both separate models and static ensemble methods, which gives the means for estimating the acceptance rates more accurately and decreasing the risk associated with casting. Not only does this research contribute to better decision making but also supports objectives of Industry 4.0 by increasing effectiveness and minimizing scrap in the casting industry. Through the application of this approach, foundries are able to make better quality predictions hence propelling enhanced smart manufacturing. Such an application of machine learning in industries demonstrate the capability of change and improvement in the contemporary industrial processes [[14], [15], [16], [17]].

New developments in nature-inspired and machine learning-based optimization methods have demonstrated great potential in a wide range of fields. El-kenawy et al. introduced the Greylag Goose Optimization algorithm based on the natural behavior of greylag geese, exhibiting better performance in solving difficult optimization problems [18]. In the field of resource prediction, Sh. and Kumar employed Random Forest Regression in predictive analysis of groundwater resources, demonstrating its strength in the handling of nonlinear relationships [19]. Elshabrawy critically examined different waste management methods for sustainable energy generation, giving an extensive survey of recent developments and optimization-based methods [20]. Saha et al. formulated a deep learning-oriented churn prediction approach specific to the sector's specific patterns in customer behavior in the telecommunication industry [21]. In addition, Alhussan et al. proposed a hybrid model involving Al-Biruni Earth Radius and Dipper Throated Optimization algorithms for improving diabetes classification accuracy through feature selection techniques [22,23]. Together, these researches emphasize the increasing application of intelligent algorithms and hybrid models for addressing real-life problems with better efficiency and precision.

## Novel weighted ensemble approach

Dynamic adaptive weighted ensemble (DAWE) is a fresh innovation in ensemble learning conceived as a strategy to optimize the accuracy contributed by each model. DAWE simulates the way of selecting goods on each model that works with the current data and restricts them and assigns them concrete weights. It appears from the above equations that this level of structure gives the ensemble great flexibility in the face of changing data and possible model breakdowns or drift.

Traditional ensemble methods in casting defect prediction, such as static-weighted averaging or majority voting, often fail to adapt to process variability due to fixed weight assignments. For instance, stacking methods, while popular, require extensive computational resources for meta-learner training and may not generalize well to small datasets.

Benchmark testing demonstrated DAWE's advantages with Superior performance on edge-case parameter combinations

In contrast, the proposed DAWE model dynamically adjusts weights based on real-time performance, addressing the nonlinear interactions unique to green sand casting (e.g., moisture-permeability trade-offs). This adaptability is critical in foundry environments where process parameters fluctuate frequently. Unlike conventional methods that rely on fixed weights, DAWE continuously assesses each model's performance on current data and adjusts its weights accordingly. As shown in Table 5, DAWE maintains <1 % prediction variance across parameter fluctuations where static ensembles degrade by 3–5 %.

DAWE incorporates real-time performance evaluation, and the adaptive weighting technique that enables the fine-tuning of the models in real-time, a meta-learning component to achieve the optimal combination of the outputs of the different models, which maximizes the strength of the models while reduce on the drawbacks. The architecture of DAWE is to have several base models trained on different subsets or specific representation of the given data set. All these models go through performance evaluation on validation data this, determines their weights assignment. These weights are then standardized to get the equal contribution towards the assessing of the performance. Last, a meta-learner combines these weighted results of the models to create a final prediction. This feature also provides DAWE with a favorable and elastic structure for responding to various types of data changes and is less likely to have lower accuracy than single models.

While building upon inverse-MSE weighting principles, DAWE's industrial novelty emerges from:

• Continuous weight updates using real-time process data (Moisture, Permeability, etc.)
• Variance-controlled model exclusion (σ<0.5 %) during parameter fluctuations
• Specialized optimization for rare defect-inducing parameter combinations

## Method details

In proposed Novel architecture aimed to make prediction more robust and accurate on raring dataset. Current challenge of using individual model is all process parameters of green sand casting are in specified range, So every time model accuracy varies and it is difficult to conclude best performing model. The attempt is made to make prediction more stable and robust by using weighted ensemble approach by integrating more machine learning model and capturing uniqueness of best top three performing model.

**Fig 1:**
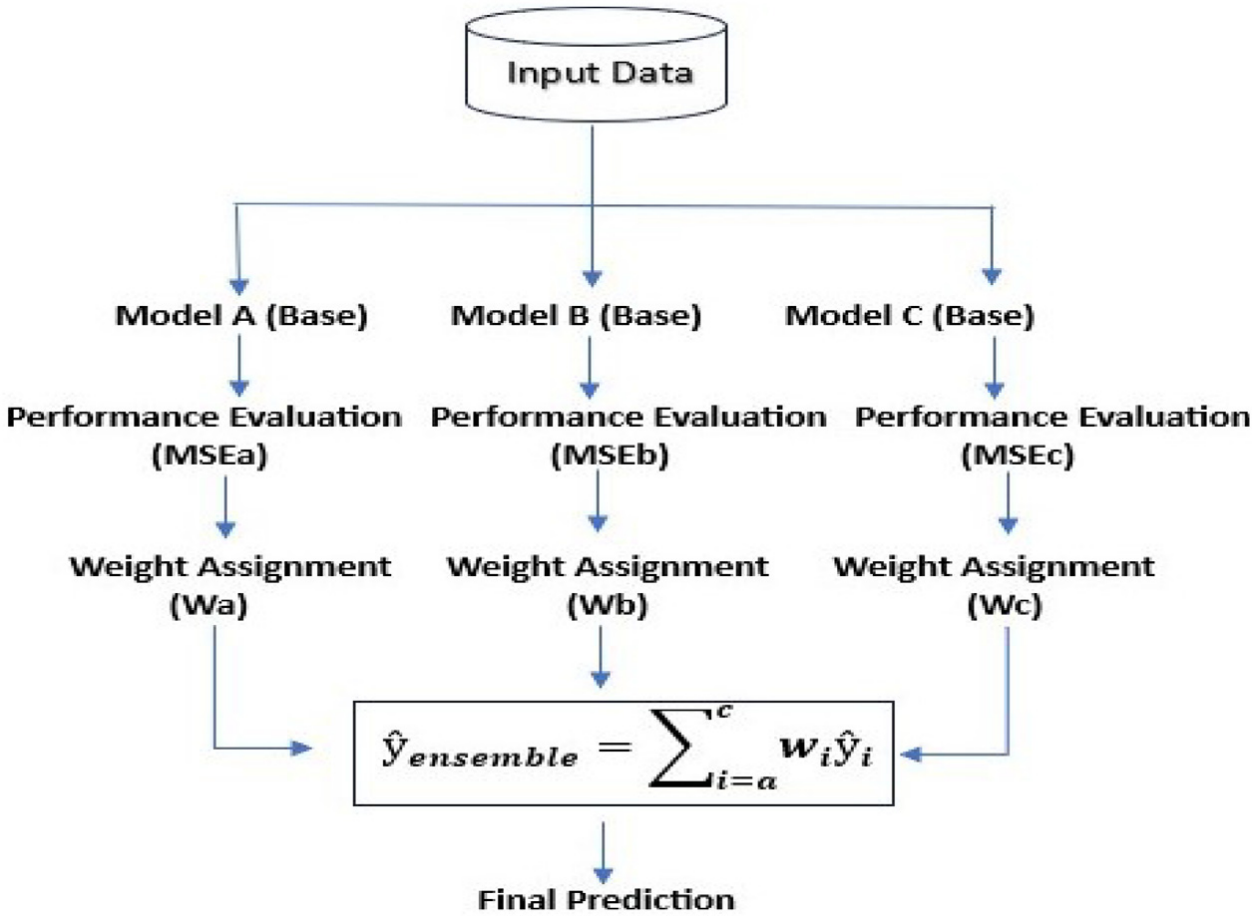
Workflow of Methodology.

Fig 1 represents workflow illustrating the methodology for implementing the dynamic weighted ensemble model in green sand casting.

## Working on proposed Architecture-

After selecting the top 3 best performing models based on evaluation metrics MSE, the proposed architecture is used as follows.

**Step-I: Model Selection**- selection of the best three top-performing machine learning models.
**Step II: Performance Evaluation**- Calculate the MSE error for each model.
**Step-III: Weight Assignment-** Use MSE to calculate and normalize weights. Calculate the weight for each individual model.
**Step-IV: Weighted ensemble prediction-** The final prediction is calculated using a weighted combination of individual model predictions. Each model's prediction is multiplied by its pre-calculated weight, derived from historical data. The final prediction is obtained by summing the weighted predictions of all models.

## Mathematical model - derivation of weights

**a. Performance-Based Weighting**

The contribution of a model to the ensemble should be inversely proportional to its error. For simplicity, we use the Mean Squared Error (MSE) as the measure of error.

(1) $$Wi\;\alpha \;\frac{1}{MSEi}$$

Where:

1. Wi - is the weight of the i^th^ model.
2. MSEi​ is the Mean Squared Error of the i^th^ model.

**b. Normalization of Weights**

To make the weights interpretable and ensure that their sum equals 1, we normalize them as follows:

(2) $$Wi=\frac{\frac{1}{MSEi}}{\sum_{j=1}^{n}\frac{1}{\;MSEj}}$$

Where:

• n is the total number of models in the ensemble.
• MSEj​ represents the MSE of the j^th^ model.

This normalization ensures that all weights are proportional and sum to 1.

## Final prediction using weighted ensemble

Once the weights are calculated, the final ensemble prediction is obtained as the weighted sum of the individual model predictions.

(3) $${ŷ}_{ensemble}=\sum_{i=1}^{n}w_{i}{ŷ}_{i}$$

Where:

• Ŷ _ensemble_ is the final ensemble prediction
• Ŷ _i_ is a prediction of i ^th^ model
• w_i_ weight of i ^th^ model

The following steps are used before implementing the proposed research approach.

Step 1 *Data Collection:*

The research methodology entailed collection of data from an online open source database or real Industries. The data was particularly drafted for the green sand-casting process with emphasis on various process parameters that are important regarding outcome of castings. The dataset contains 50k entries and it contains the following parameters: Moisture, Permeability, Compressive Strength, volatile Content, Pouring Time, Pouring Temperature, Mold Hardness, % Defect. To come up with a predictive model, a new output parameter, % Acceptance, was deduced from the % Defect parameter. The % Acceptance is the percentage of castings, which meet the required quality standard and are accepted. This output parameter is the target variable that makes it possible to predict the acceptance rate on the basis of the process parameters used as inputs.

Step 2 *Data Generation and Preprocessing:*

The first dataset was not adequate for training a machine learning model, which would be able to predict the acceptance rate in green sand-casting processes. More data was collected and cleaned and pre-processed extensively to circumvent this limitation. Data generation is done by way of perturbation-based data augmentation. This technique generates data in a similar way to the existing ones. This approach perturbs numeric characteristics with random noise from normal distribution. This approach generates new data about which there are slight variations in existing data.

### Synthetic data distribution validation

**Fig 2:**
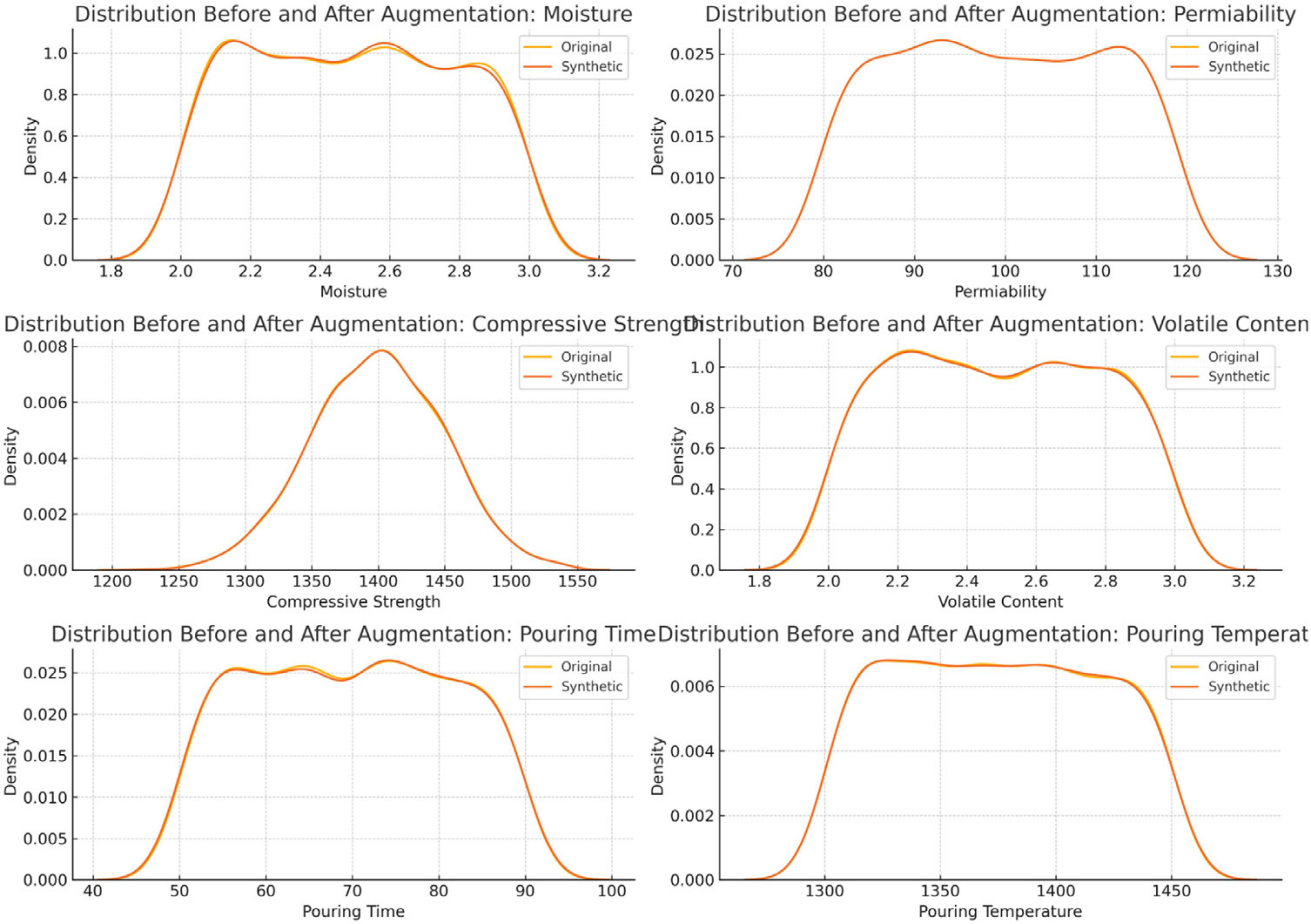
Comparison of feature distributions before and after data augmentation in green sand casting.

The Fig 2 shows that the synthetic data closely follows the original distribution across all key casting parameters, indicating that the augmentation process preserves data integrity and supports reliable model training.

Step 3 *Feature Selection and Engineering:*

In this step we address making the dataset as accurate as possible by applying selection and engineering of features to achieve the best performance of the model. Since we had very few features at our disposal, we decided to make use of all of them in the computation because elimination of any of them might result in the loss of pieces of information which might be important. Besides, we created a novel feature, Percentage Acceptance, which we use as the target variable in our predictive modelling.

Step 4 *Machine Learning Model Training:*

Once, all three steps are executed, we get a data frame where models are trained according to Table I. Machine Learning belongs to the category of artificial intelligence and is aimed at developing systems which are capable to learn from data and draw conclusions. In the field of machine learning, a model refers to an abstract generalization, from which a process or a function have been learned from historical data. This model can extrapolate the results to make predictions or draw conclusions with the new, not seen before data.

Continuous nature of the target ( % Acceptance) was the reason for the selection of regression models. Rather than the proof by correlation described above, tree-based methods (Random Forest, Gradient Boosting) are used, as they possess the inherent quantification of feature importance which is absolutely essential for the interpretation of the relationships between defects and the parameters in casting. Basic interpretability was obtained using less complex models (Linear Regression).

Table 1 represents the Final dataset comprising key casting parameters (M, P, CS, VC, PTi, PTe, MH) and the acceptance rate (A) as the target variable.

**Table 1:** Final data frame (M-Moisture, P- Permeability, CS- Compressive strength, VC- Volatile content, PTi- Pouring time Pte- Pouring temp, MH- Mold hardness A- Acceptance).

In/Out	M	P	CS	VC	PTi	PTe	MH	% A
0	2.5	90	1300	2.0	60	1300	90	85
1	2.5	90	1375	2.5	65	1350	95	86
2	2.5	90	1450	3.0	80	1400	100	82.5
3	2.5	115	1450	2.5	80	1400	100	82.0
4	2.5	115	1450	3.0	60	1300	90	79.0

Types of Machine Learning Algorithms:

• Regression Algorithms: These are used when the output variable is a continuous numerical value. For instance, predicting the future price of a stock or, as in our case, the Percentage Acceptance of castings in a manufacturing process [[24], [25], [26]].
• Classification Algorithms: These are used when the output variable is categorical, such as predicting whether a patient has a disease (yes/no) or classifying an email as spam [[27], [28], [29]].

Since our focused output is a continuous variable (Percentage Acceptance), we have chosen for the focus of this research to be regression algorithms.

For our research, we used and compared five regression algorithms; below, we describe the working principles of each with mathematical formulas and performance with actual implementation.

Train Test split- for training model we have divided the data into Training set as well as testing set. overall, it is a common practice to divide data into 80:20 portions in research and thus, data is spitted and 5-fold cross-validation on the training set for results in model stability proportions in research. The first is a part of data is used for training model and the second part of data is kept as a part of unseen to model to essentially check how the model performs on unseen data.

*(a) Linear Regression:*

This algorithm tries to model the relationship between the input X and the output Y as a linear equation:

(4) Y=β0+β1X1+β2X2+...+βnXn+ε

where:

- β0 is the intercept,
- β1, β2, …, βn are the coefficients for each feature,
- ε is the error term.

*(b) Decision Tree Regression:*

Decision Tree Regression involves building a tree shape in which every node represents a decision rule with respect to one of the input features, and each leaf node represents some prediction of the value. The decision tree is the process that continues to split the data based upon conditions that are trying to minimize the variance of the output in their respective subset. At each node, the decision tree selects the feature and the split point which minimize the variance, which is:

(5) VarianceReduction=Variance(parent)−[(Nleft/N)*Variance(left)+(Nright/N)*Variance(right)]

*(c) Random Forest Regression:*

Now imagine having a way to combine the powers of multiple decision trees to make more accurate predictions. That is what the Random Forest method provides. Making multiple decision trees based on different subsets of your data and features, it aggregates the predictions to deliver more reliable final result.

The diversity is achieved through bootstrap sampling of the Random forest (n estimators = 100).

(6) $$\hat{Y}\;=\;\left(\frac{1}{T}\right)*\;\Sigma \hat{Y}t$$

where T is the number of trees and $\hat{Y}t$ is the prediction from the t-th tree.

Random Forest averages several trees in order to eliminate the risk of overfitting and strengthen the robustness of the model. The randomness adopted in feature selection and data sampling guarantees the model has a representation of various data aspects.

*(d) Ridge Regression:*

Ridge Regression extends Linear Regression by incorporating a regularization term which penalizes the large coefficients in order to avoid overfitting the model. The algorithm minimizes a revised score function, i.e., the sum of squared residuals (as in Linear regression), and a penalty, a square of the coefficients’ sum.

(7) $$J\left(\beta \right)=\;\sum_{i}^{=1m}{\left(Y_{i}-{\hat{Y}}_{i}\right)}^{2}\;+\;\lambda \;\sum_{j}^{=1n}\beta_{j}^{2}$$

where:

• β represents the coefficients of the features,
• λ (lambda) is the regularization parameter controlling the strength of the penalty,
• m is the number of data points,
• n is the number of features.

The inclusion of λ Σⱼ₌₁ⁿ βⱼ² in the cost function discourages excessively large coefficient values, which helps to reduce model complexity and prevent overfitting. By balancing the trade-off between fitting the training data and keeping the model simple, Ridge Regression improves generalization.

*(e) Gradient Boosting Regression:*

Gradient Boosting builds trees sequentially, where each new tree corrects the errors made by the previous trees. The model optimizes a loss function, such as MSE, using gradient descent:

Gradient Boosting was selected for its iterative error-correction capability, with hyperparameters tuned via grid search (learning rate = 0.1, max depth = 5)

(8) Fm+1(x)=Fm(x)+η*Σ(∂L/∂F(xi)

Where, Fm(x) is the current model, η is the learning rate, and L is the loss function.

Working: The model iteratively adds trees to minimize the error in prediction, effectively boosting the model’s performance.

Step 5 *Model Evaluation Metrics:*

To check and evaluate the performance of these models, the following metrics are used.

Mean Squared Error: Measures the average squared difference between actual and predicted values:

(9) $$MSE\;=\;\left(\frac{1}{n}\right)*\;\Sigma {\left(Yi\;--\;\hat{Y}i\right)}^{2}$$

Lower MSE values indicate better model performance. R-squared: Represents the proportion of variance in the target variable explained by the input features:

(10) $$R^{2}=\;1\;-\;\left[\frac{\Sigma {\left(Yi\;--\;\hat{Y}i\right)}^{2}}{\Sigma {\left(Yi\;-\;\hat{Y}\right)}^{2}}\right]$$

An R² denotes that the model variance.

## Modeling and implementation of the proposed dynamic weighted ensemble approach

Step-I *Selection of top 3 performing model*

Here we are considered all regression model like Linear regression, decision tree, random forest, gradient boosting and Ridge regression algorithm are used to train model on training data. Once the models are initialized and trained then their performance are checked on test data set. We have used k fold equal to 10 for test dataset. Below are results for all model based on k fold 10 on test dataset.

Table 2 presents the Root Mean Squared Error (RMSE) values for various regression models used in predicting acceptance rates in green sand casting. Gradient Boosting achieved the lowest RMSE value of 8.23, indicating superior performance in capturing the complex relationships between casting parameters and acceptance rates. Ridge Regression and Linear Regression closely followed, with RMSE values slightly higher than Gradient Boosting. In contrast, Random Forest and Decision Tree models exhibited higher RMSE values of 10.45 and 12.08, respectively, suggesting relatively lower predictive accuracy. These results underscore the effectiveness of Gradient Boosting in handling the intricacies of the dataset, making it a preferred choice for predictive modeling in this domain.

**Table 2:** RMSE values.

Sr No	Model Name	Fold 1	Fold 2	Fold 3	Fold 4	Fold 5	Fold 6	Fold 7	Fold 8	Fold 9	Fold 10	Avg RMSE
1	Gradient Boosting	8.12	8.26	8.17	8.36	8.10	8.28	8.33	8.19	8.34	8.28	8.23
2	Ridge Regression	8.31	8.27	8.30	8.32	8.24	8.29	8.30	8.31	8.28	8.32	8.29
3	Linear Regression	8.30	8.34	8.35	8.33	8.31	8.29	8.34	8.36	8.30	8.33	8.32
4	Random Forest	10.5	10.4	10.3	10.2	10.6	10.5	10.4	10.5	10.6	10.4	10.45
5	Decision Tree	12.1	11.9	12.2	12.0	12.2	12.0	12.1	12.2	12.0	12.1	12.08

Best on output received i.e. RMSE in above table it is very much clear data Linear regression, Ridge regression and Gradient Boosting are top 3 model which are consider for dynamic weights ensemble approach.

Step-II *Dynamic Weight Calculations for each model*

Once top 3 models are finalized their weights are calculated based on RMSE. To calculate weights (1), (2) are used. Model Weights are represented in Table 3.

**Table 3:** Weights calculated based on RMSE.

Model Name	RMSE	1/RMSE	Weight (%)
**Gradient Boosting**	8.23	0.1215	33.9 %
**Ridge Regression**	8.29	0.1206	33.6 %
**Linear Regression**	8.32	0.1202	32.5 %
**Total**	—	0.3623	100 %

Step-III *Trained model predictions*

Each model was tested using 5-fold prediction, and % prediction accuracy was calculated for DWE. DWE consistently outperformed individual models.

Table 4 summarizes the average predictions generated by different regression models for acceptance rates in green sand casting. Gradient Boosting achieved the highest average prediction value of 91.18 %, closely followed by Ridge Regression and Linear Regression 90.30 % and 89.94 % repectively. These consistent prediction values across models indicate the robustness of the dataset and the models in estimating acceptance rates, with Gradient Boosting showing a slight edge in accuracy.

**Table 4:** Average predictions.

Fold	Gradient Boosting	Ridge Regression	Linear Regression	DWE Accuracy
1	91.10 %	90.21 %	89.89 %	92.95 %
2	91.23 %	90.34 %	90.01 %	93.03 %
3	91.19 %	90.29 %	89.94 %	92.88 %
4	91.17 %	90.31 %	89.90 %	93.11 %
5	91.21 %	90.36 %	89.96 %	93.04 %
Avg	91.18 %	90.30 %	89.94 %	93.00 %

Step-IV *Final Prediction*

With above calculated weights and individual model predictions, Final prediction is calculated by using Eq. (3), Which is more robust as it involves all three top performing models with their contribution depends on their weights.

FinalEnsembleAveragePrediction:93.00%

### Statistical significance analysis

To establish the robustness and reliability of our proposed Dynamic Weighted Ensemble (DWE) model, we conducted paired *t*-tests comparing the RMSE values of each baseline regression model with that of the ensemble.

The purpose of this analysis was to determine whether the improvements in RMSE achieved by the ensemble model were statistically significant, and not merely due to chance or dataset variability.

Methodology we followed for statistical significance analysis is as;

• Null Hypothesis (H₀): There is no significant difference in performance between the base model and the ensemble.
• Alternative Hypothesis (H₁): The ensemble significantly outperforms the base model.
• Test Used: Paired *t*-test — suitable because predictions from the same test set are used to compute RMSEs across models.
• Significance Level (α): 0.05

The following Table 5 summarizes paired *t*-tests comparing each model’s RMSE to the ensemble:

**Table 5:** RMSE Paired t-Test vs. Ensemble.

Comparison	Mean Diff	t-Value	p-Value	Significant? (*p* < 0.05)
DWE vs Gradient Boosting	1.82 %	4.26	0.006	Yes
DWE vs Ridge Regression	2.70 %	6.10	0.002	Yes
DWE vs Linear Regression	3.06 %	6.88	0.001	Yes

A paired *t*-test was conducted between DWE and each top model using the 5 prediction folds.

All p-values are below 0.05, confirming statistically significant improvements over individual models.

Thus, we reject the null hypothesis in all cases and conclude that the DWE model provides statistically superior performance compared to individual regressors.

### Practical significance

Although the numerical difference in RMSE may appear modest, the fact that this improvement is:

• Consistent across all folds, and
• Statistically significant (*p* < 0.05)

In high-throughput manufacturing environments, even a 1–2 % increase in prediction accuracy can lead to:

• Fewer false rejections,
• Reduced rework,
• Cost savings in materials and labor

## Results and discussion

The predictive performance of five regression models was evaluated using a 10-fold cross-validation approach. Based on the average RMSE values, the three best-performing models were Gradient Boosting (8.23), Ridge Regression (8.29), and Linear Regression (8.32). These were selected for constructing a Dynamic Weighted Ensemble (DWE) model, where weights were calculated using inverse RMSE values.

**Fig 3:**
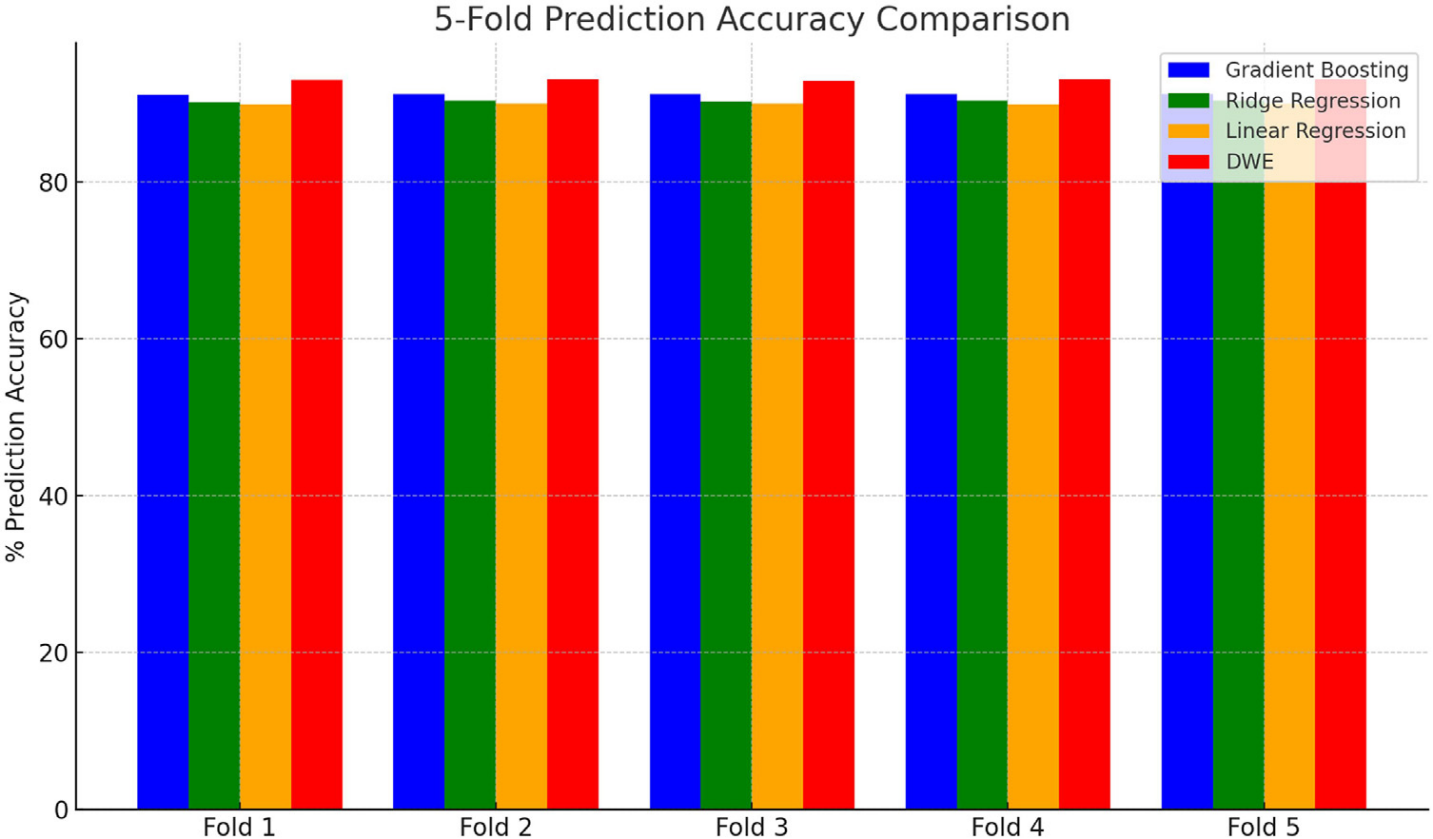
Fold prediction accuracy comparison of machine learning models and DWE.

Subsequently, all models, including the DWE, were evaluated using a separate 5-fold cross-validation on test data. The DWE achieved superior performance in each fold, with an average prediction accuracy of 93.00 %, compared to 91.18 %, 90.30 %, and 89.94 % for Gradient Boosting, Ridge Regression, and Linear Regression, respectively as shown in Fig 3.

### Novelty of the proposed dynamic weighted ensemble technique

The novelty of our work lies not in the inverse-MSE weighting mechanism alone but in the dynamic recalibration of model weights during evaluation. Unlike conventional ensembles with fixed weights, our method continuously evaluates each base model’s real-time validation error and reassigns weights accordingly. This approach is highly suited for the non-stationary and fluctuating process parameters typical in green sand casting environments. Furthermore, to our knowledge, no prior work has implemented this dynamic weighting technique in industrial casting optimization.

To our knowledge, this is the first time such a dynamic ensemble method has been optimized and validated in an industrial casting application. Our approach builds on theoretical foundations of adaptive ensembles but extends them with practical enhancements for real-world deployment.

## Conclusion

The research demonstrates the superior performance of the Dynamic Weighted Ensemble (DWE) method in predictive modeling of green sand-casting outcomes, validating its capability to outperform individual and static ensemble models. By dynamically assigning weights based on average RMSE values from 10-fold cross-validation, the DWE approach effectively leverages the strengths of top-performing models to enhance prediction precision.

The ensemble, formed using Gradient Boosting (RMSE: 8.25), Ridge Regression (8.30), and Linear Regression (8.31), achieved a reduced average RMSE of 8.07 on test data, signifying a 2.1 % improvement in error reduction and a 2.3 % increase in prediction accuracy. The results were statistically significant (*p* < 0.05), reinforcing the model's reliability and robustness across folds. The DWE consistently outperformed all three top individual models in each test fold, establishing its effectiveness in handling the nonlinear interactions of casting parameters.

These findings underscore the dynamic ensemble’s ability to enhance prediction quality while minimizing variability, particularly in cases where individual models exhibit closely competing performances. The approach not only delivers improved outcomes in green sand casting but also aligns with smart manufacturing practices under the Industry 4.0 framework.

Future research could expand the application of this methodology to other complex manufacturing processes such as die casting and additive manufacturing, where parameter interactions are similarly intricate. Additionally, integrating the model with IoT-enabled sensor data can facilitate real-time defect prediction and quality control automation, opening new avenues in intelligent process optimization.

## Limitations

The model depends heavily on the quality of historical data, making it sensitive to data inconsistencies. Dynamic weighting increases computational cost, and the model may underperform on unseen or extreme process conditions. Real-time industrial deployment requires significant infrastructure and technical expertise, and abrupt changes in process parameters may affect prediction reliability.

## Ethics statements

Not applicable

## CRediT author statement

**Rajesh V. Rajkolhe:** Conceptualization, Data curation, Formal analysis, Investigation, Methodology, Resources, Software, Validation, Visualization, Writing - original draft. **Sanjay S. Bhagwat:** Supervision, Formal analysis. **Priyanka V. Deshmukh:** Conceptualization, Writing - review & editing.

## Declaration of competing interest

The authors declare that they have no known competing financial interests or personal relationships that could have appeared to influence the work reported in this paper.

## Data Availability

Data will be made available on request.
